# Interleukin-1 Receptor Antagonist (IL-1RA) Mediates Age-Dependent Immunoregulation of Adipose-Derived Stem Cells via Macrophage Polarization

**DOI:** 10.3390/cells15151355

**Published:** 2026-07-28

**Authors:** Qiuling Dong, Yu Zhu, Shisan Xu, Dijie Li, Qiong Wu

**Affiliations:** Guangxi Universities Key Laboratory of Stem Cell and Biopharmaceutical Technology, Research Center for Biomedical Sciences, School of Life Sciences, Guangxi Normal University, Guilin 541004, China; 14795988965@163.com (Q.D.); zhuyu1813@outlook.com (Y.Z.); perseverancexss@163.com (S.X.);

**Keywords:** adipose-derived stem cells, IL-1RA, macrophage polarization, immunomodulation, aging, IL-1β

## Abstract

This study aimed to clarify whether interleukin-1 receptor antagonist (IL-1RA) mediates the age-dependent immunomodulatory capacity of adipose-derived stem cells (ASCs) via regulating macrophage polarization. ASCs from young (Y-ASCs) and aged (O-ASCs) C57BL/6 mice were isolated. IL-1RA, which was identified as an age-sensitive candidate through integrated transcriptomic and proteomic screening in our prior publication, was validated by qPCR, immunofluorescence, and ELISA. RAW264.7 macrophages were polarized and co-cultured with ASCs or treated with recombinant IL-1RA; IL-1RA in Y-ASCs was silenced by siRNA (82.3 ± 5.1% knockdown efficiency). The results showed Y-ASCs had 2.1-fold higher IL-1RA mRNA and significantly higher protein secretion than O-ASCs (*p* < 0.001). Y-ASC transplantation enhanced M2 polarization (CD206^+^ cells increased from 9.36% to 21.4% in vivo; *p* < 0.01) in aged mouse adipose tissue and reduced inflammation (serum IL-1β lowered by 42.3 ± 5.1%; *p* < 0.001), while O-ASCs had no effect. Recombinant IL-1RA recapitulated Y-ASC effects (M2 macrophages increased from 0.92% to 61.8%; *p* < 0.001), and IL-1RA silencing abrogated Y-ASC function. The data indicate IL-1RA is a key age-sensitive mediator of ASC immunomodulation. Declined IL-1RA may contribute to impaired aged ASC efficacy, highlighting donor age’s importance. This provides mechanistic insights and guides IL-1RA-targeted optimization for ASC therapies in age-related inflammatory disorders.

## 1. Introduction

Aging is a major risk factor for metabolic disorders such as obesity and type 2 diabetes, with the dysregulation of adipose tissue immune microenvironment emerging as a central pathological driver [1]. Adipose tissue macrophages play a pivotal role in this process, with the accumulation of macrophages and their skewing toward the pro-inflammatory M1 phenotype triggering chronic low-grade inflammation (inflammaging) [2,3], which disrupts adipose tissue homeostasis and exacerbates metabolic dysfunction. In contrast, anti-inflammatory M2 macrophages support tissue repair and metabolic balance [4], making the modulation of macrophage polarization a promising therapeutic target for age-related metabolic and inflammatory disorders.

Adipose-derived stem cells (ASCs) have garnered significant attention in regenerative medicine and immunotherapy due to their inherent immunomodulatory capacity, primarily exerted via paracrine secretion of bioactive factors. Mounting evidence indicates that ASCs can skew macrophage polarization from M1 to M2, suppress pro-inflammatory cytokine production, and alleviate chronic inflammation in various disease models [5,6]. However, ASC therapeutic efficacy is highly heterogeneous, with donor age identified as a critical confounding factor—young ASCs (Y-ASCs) consistently outperform aged ASCs (O-ASCs) in preclinical studies [7,8], aligning with our preliminary observations that aging-related functional alterations of ASCs [9] and Y-ASC transplantation effectively reduce body weight in aged mice, while O-ASC transplantation fails to elicit such effects [10]. Despite this well-documented age-dependent functional decline, the key molecular mediators and underlying mechanisms remain incompletely elucidated.

The interleukin-1 (IL-1) signaling pathway is a well-established master regulator of innate inflammation. The pro-inflammatory cytokine IL-1β and its endogenous antagonist IL-1RA form a critical signaling axis that maintains immune homeostasis, while dysregulation of this axis promotes tissue dysfunction and cellular senescence [11,12]. Previous studies have linked IL-1RA to macrophage polarization and inflammatory resolution, and its expression is dynamically regulated during aging [13,14]. Adipose-derived stem cells (ASCs) hold great promise for treating inflammatory disorders, yet their immunomodulatory potency is known to decline with donor age [15]. The molecular drivers of this age-related functional impairment remain poorly defined. Among the key paracrine mediators of mesenchymal stem cell action, interleukin-1 receptor antagonist (IL-1RA) has been demonstrated to be crucial for their anti-inflammatory effects [14,16] and for reprogramming activated macrophages toward a regulatory, M2-like phenotype [17]. In our prior transcriptomic and proteomic screening, IL-1RA emerged as an age-sensitive candidate in ASCs. Given the established role of IL-1RA in macrophage biology and the age-dependent decline in ASC paracrine function, we hypothesized that the age-dependent decline in IL-1RA secretion by ASCs impairs their ability to promote M2 macrophage polarization, thereby diminishing their immunomodulatory capacity.

To test this hypothesis, we systematically investigated IL-1RA’s role in ASC-mediated macrophage polarization in vitro and in vivo. Specifically, we compared IL-1RA expression levels between Y-ASCs and O-ASCs, evaluated IL-1RA’s impact on ASC-induced macrophage subset remodeling, and validated the causal contribution of IL-1RA to ASC function via gain-of-function and loss-of-function experiments. This study aims to fill the mechanistic gap in age-dependent ASC immunomodulation by investigating IL-1RA as a key mediator, and to provide novel insights for optimizing ASC-based therapies for age-related inflammatory conditions, particularly for allogeneic ASC therapy donor selection.

## 2. Materials and Methods

### 2.1. Animals and ASC Isolation

SPF-grade male C57BL/6 mice (young: 2 months, initial body weight 20–22 g; aged: 18–20 months, initial body weight 32–34 g) were housed in the SPF-grade animal facility of Guangxi Normal University under specific pathogen-free conditions with a 12 h light/dark cycle, ad libitum access to standard chow and water. All mice were acclimatized for 7 days under the same housing conditions before formal experiments. All animal procedures were approved by the Institutional Animal Care and Use Committee of Guangxi Normal University (protocol code GXNU-202501-008), with preset humane endpoints (≥20% baseline body weight loss, moribund state) for euthanasia to minimize animal suffering.

Adipose-derived stem cells (ASCs) were isolated from inguinal subcutaneous adipose tissue of young and aged male C57BL/6 mice. Mice were euthanized, and fat pads were excised under aseptic conditions. Tissues were minced and digested in 0.075% collagenase type I (Sigma-Aldrich, St. Louis, MO, USA) at 37 °C for 45 min with constant shaking. The digested mixture was filtered through a 70 µm nylon mesh and centrifuged at 1200× *g* for 10 min to collect the stromal vascular fraction. Cells were resuspended in DMEM/F12 (Gibco, Waltham, MA, USA) supplemented with 10% fetal bovine serum (FBS, Gibco) and 1% penicillin–streptomycin, and cultured at 37 °C in 5% CO_2_. Cells at passages 3–5 were used for experiments. ASC identity and quality were validated according to standard criteria. Flow cytometric analysis confirmed a canonical mesenchymal stem cell surface phenotype (CD29^+^, CD44^+^, CD105^+^, CD45^−^). The multilineage differentiation capacity, proliferation rate, viability and senescence status of Y-ASCs and O-ASCs at passages 3–5 have been systematically characterized under strictly matched culture conditions in our previous work [9,14], confirming that the observed functional differences are not confounded by cell viability or culture adaptation.

### 2.2. Macrophage Culture and Polarization

RAW264.7 murine macrophages were cultured in DMEM containing 10% FBS at 37 °C. To induce polarization, cells were treated with lipopolysaccharide (LPS, 1 μg/mL, Sigma) for M1 polarization or interleukin-4 (IL-4, 40 ng/mL, PeproTech, Rocky Hill, NJ, USA) for M2 polarization for 24 h. Using LPS alone to induce M1 polarization is a commonly reported practice in published ASC–macrophage co-culture studies [17]. For co-culture studies, macrophages were cultured in Transwell systems (0.4 µm pore size, Corning, Corning, NY, USA) with ASCs seeded in the upper chamber. In cytokine stimulation experiments, macrophages were treated with recombinant mouse IL-1RA (100 ng/mL, R&D Systems, Minneapolis, MN, USA) for 24 h. This dose was selected based on published studies demonstrating effective M2 polarization at this concentration and matches the physiological range of IL-1RA secreted by Y-ASCs in our co-culture system [18,19].

### 2.3. Quantitative PCR Analysis

Total RNA was extracted using TRIzol reagent (Invitrogen, Carlsbad, CA, USA) according to the manufacturer’s instructions. cDNA was synthesized using a PrimeScript RT reagent kit (Takara, Kusatsu, Shiga, Japan). Quantitative real-time PCR was performed using SYBR Green Master Mix (Bio-Rad, Hercules, CA, USA) on a CFX96 Touch Real-Time PCR system (Bio-Rad). Relative mRNA levels were calculated using the 2^^−ΔΔCt^ method and normalized to *Gapdh* expression. The primer sequences are shown in Table S1.

### 2.4. Immunofluorescence

ASCs were fixed in 4% paraformaldehyde for 15 min, permeabilized with 0.1% Triton X-100, and blocked with 5% BSA. Cells were incubated with anti-IL-1RA antibody (1:200, Proteintech, 10844-1-AP, Rosemont, IL, USA) overnight at 4 °C, followed by Alexa Fluor-conjugated secondary antibodies. Nuclei were counterstained with DAPI. All images were captured under identical exposure settings using a Leica DMi8 (Leica Microsystems, Wetzlar, Germany) fluorescence microscope. For quantification, at least 5 random fields per sample were analyzed, with background fluorescence subtracted using ImageJ (U.S. National Institutes of Health, Bethesda, MD, USA) software. Image analysis was performed by a blinded investigator.

### 2.5. Flow Cytometry

For surface marker analysis, macrophages were stained with anti-F4/80-FITC (BM8, BioLegend, 123108, San Diego, CA, USA), anti-CD206-APC (C068C2, BioLegend, 141708), anti-CD11c-PE-rFlour (N418, BioLegend, 128710) and anti-CD11b-PE-Cy7 (M1/70, BioLegend, 101216) at 1:200 dilution for 30 min at 4 °C in the dark. Cells were gated sequentially for singlets, live cells, and F4/80^+^CD11b^+^ macrophages before subset analysis. Fluorescence minus one (FMO) controls were used to set positive gates. After washing, cells were analyzed using a BD FACSCalibur cytometer (BD Biosciences, San Jose, CA, USA), with at least 10,000 events acquired per sample. Data were analyzed using FlowJo (BD Life Sciences, Ashland, OR, USA) software.

### 2.6. siRNA Transfection

IL-1RA expression in Y-ASCs was knocked down using IL-1RA-targeted siRNA (GenePharma, Shanghai, China) with Lipofectamine RNAiMAX (Invitrogen) according to the manufacturer’s protocol. A scrambled siRNA served as the negative control. Knockdown efficiency was confirmed by qPCR and ELISA. The siRNA oligonucleotide sequences used in this study were as follows: sense strand, 5′-GGAUGGUUCCUCUGCACAATT-3′; antisense strand, 5′-UUGUGCAGAGGAACCAUCCTT-3′.

### 2.7. In Vivo Transplantation and ELISA

ASCs at passage 3 were digested with trypsin (Thermo Fisher Scientific, Waltham, MA, USA), centrifuged at 300× *g* for 5 min and resuspended in phosphate-buffered saline (PBS) to a final concentration of 1 × 10^6^ ASCs in 0.2 mL. All ASC transplantations were performed as allogeneic therapy.

A total of 20 male mice were used: fifteen old mice were divided into three groups (*n* = 5 each) via body weight-stratified randomization with random number tables—PBS (Old), old ASC transplantation (O-Trans), and young ASC transplantation (Y-Trans)—while 5 young mice served as the Young control group receiving PBS (Sangon Biotech, Shanghai, China). No dedicated control strategies were implemented to mitigate potential confounders, including animal cage location, treatment administration order and sample measurement order. Full-process blinding was implemented: group allocation, reagent preparation, sample detection and data analysis were all performed by blinded investigators. Daily health monitoring was conducted for all animals throughout the experiment. Investigators performing subsequent assays and data analysis were blinded to group assignment. No pre-defined inclusion or exclusion criteria for animals, experimental units and data points were established a priori. No animals or data points were excluded during the study. Thirty days after transplantation, blood samples were collected by retro-orbital puncture, and serum IL-1β levels were measured using mouse IL-1β ELISA kits (R&D Systems), according to the manufacturer’s instructions. Adipose tissue was homogenized and collagenase-digested, stained with antibodies and analyzed by flow cytometry.

### 2.8. The Transcriptomics Analysis

Public transcriptomic datasets GSE125170 (human ASCs from normal-weight and obese donors) and GSE48964 (murine ASCs) were retrieved from the NCBI Gene Expression Omnibus (GEO) database. In-house transcriptomic data of Y-ASCs and O-ASCs were obtained from our previously deposited dataset in the Genome Sequence Archive (accession number: CRA008155). Raw read counts were normalized to transcripts per million (TPM) for gene expression quantification. Batch effects across datasets were corrected using the ComBat algorithm prior to cross-dataset comparison. Differential expression analysis was performed with Benjamini–Hochberg adjusted *p*-values. Genes with |log2 fold change| > 1 and adjusted *p* < 0.05 were defined as differentially expressed genes.

### 2.9. Data Processing

The results are reported as means ± SD, with n representing biological replicates. The sample size of in vivo experiments was determined based on the effect size of our previously published ASC transplantation study in aged mice [10]; for in vitro experiments, the sample size was set in accordance with standard cell biology experimental norms with no less than 3 independent biological replicates. No formal a priori sample size calculation was performed. Normality was tested using the Shapiro–Wilk test, and variance equality was tested using Levene’s test. All data included in this study met the assumptions of normality and equal variance; thus, parametric tests were uniformly adopted for statistical analysis. Comparisons between two groups were performed using two-tailed Student’s *t*-test. Multi-group comparisons were performed using one-way ANOVA followed by Tukey’s post hoc test for multiple comparisons. *p* < 0.05 was considered statistically significant. All statistical analyses were performed using GraphPad Prism (Dotmatics, San Diego, CA, USA). Effect sizes (Cohen’s d for two-group comparisons, partial η^2^ for multi-group comparisons) with 95% confidence intervals were calculated for all between-group comparisons.

## 3. Results

### 3.1. Macrophage Infiltration and Polarization Patterns in Adipose Tissue of Aged Mice Post-Transplantation with Y-ASCs and O-ASCs

**Figure 1:**
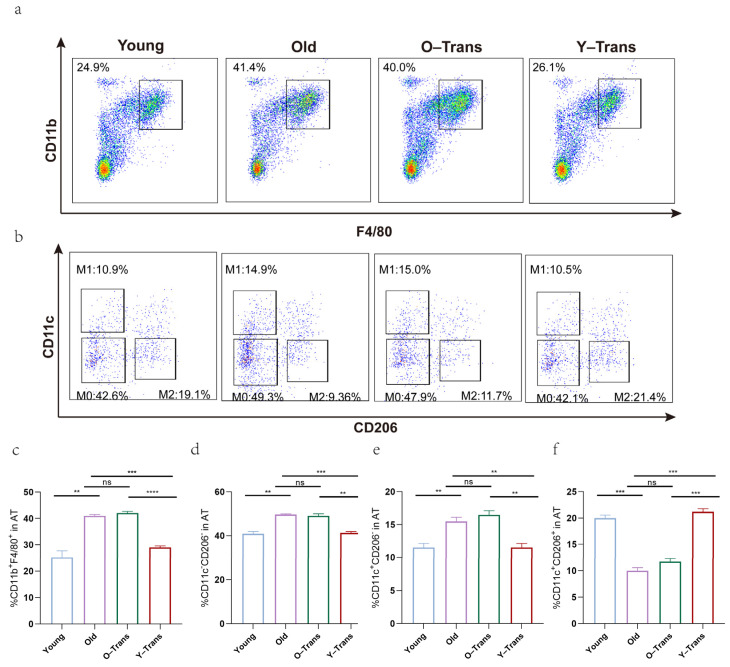
Effects of Y-ASCs and O-ASCs on adipose tissue macrophages in aged mice. (**a**,**b**) Flow cytometric analysis of macrophage abundance and polarization in adipose tissue after ASC transplantation. F4/80^+^CD11b^+^ cells were defined as total macrophages, which were subdivided into M0 (CD11c^−^CD206^−^), M1 (CD11c^+^CD206^−^), and M2 (CD11c^−^CD206^+^) subsets based on CD11c and CD206 expression. (**c**–**f**) Quantitative analysis of the proportions of M0, M1, and M2 macrophages and the abundance of total macrophages. Data are presented as the mean ± SD (*n* = 5). Significance: **** *p* < 0.0001, *** *p* < 0.001, ** *p* < 0.01, ns: not significant.

In our previously published study, we compared Y-ASCs and O-ASCs and characterized cellular aging in the ASCs themselves. The senescence-associated β-galactosidase staining, *p16*, *p21*, *p53* and SASP markers confirmed that O-ASCs were in a senescent state compared to Y-ASCs (Figure S1). Building on our previous finding that transplantation of young adipose-derived stem cells (Y-ASCs) effectively reduces body weight in aged mice, whereas aged ASC (O-ASC) transplantation does not [10], we further explored the underlying immunomodulatory mechanisms linking ASC transplantation to metabolic benefits by analyzing macrophage infiltration and polarization patterns in adipose tissue of aged mice post-transplantation. The flow cytometric analysis of F4/80^+^CD11b^+^ showed that Y-ASC transplantation significantly reduced the total abundance of macrophages in adipose tissue (from 41.4 ± 3.2% to 26.1 ± 2.8%; *p* < 0.01), while O-ASC transplantation had no significant impact on macrophage abundance compared with the control group (40.0 ± 3.5%; *p* > 0.05; Figure 1a). More importantly, macrophage subtyping revealed that Y-ASC transplantation altered the polarization balance: the proportion of pro-inflammatory M1 macrophages decreased from 14.9 ± 1.8% to 10.5 ± 1.2% (*p* < 0.05), and the proportion of anti-inflammatory M2 macrophages increased from 9.36 ± 1.5% to 21.4 ± 2.1% (*p* < 0.01; Figure 1b). Quantitative analysis confirmed a 36.2 ± 4.3% reduction in the M1/M2 ratio (*p* < 0.01) (Figure 1c–f). These in vivo findings indicate that Y-ASCs outperform O-ASCs in remodeling the age-associated inflammatory microenvironment of adipose tissue, which may underlie their superior capacity to improve metabolic phenotypes in aged mice.

### 3.2. Y-ASCs Promote M2 Macrophage Polarization More Potently than O-ASCs In Vitro

**Figure 2:**
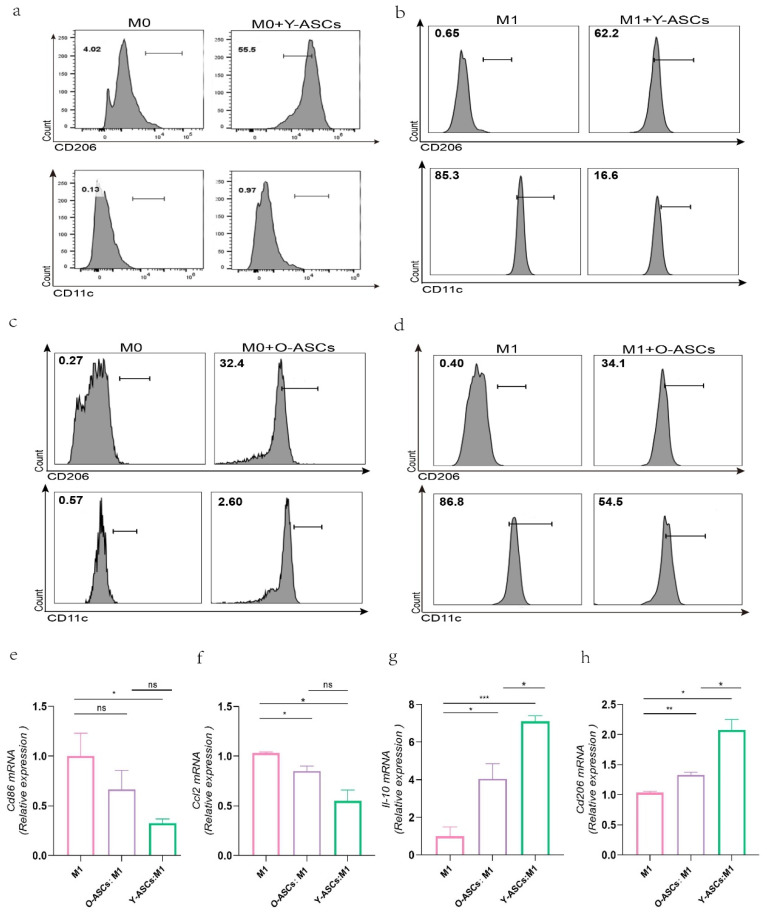
Effects of Y-ASCs and O-ASCs on macrophage polarization in vitro. (**a**,**b**) Flow cytometric analysis of CD206 and CD11c expression in M0 and M1 macrophages co-cultured with Y-ASCs. (**c**,**d**) Flow cytometric analysis of CD206 and CD11c expression in M0 and M1 macrophages co-cultured with O-ASCs. (**e**–**h**) qPCR analysis of M1 marker genes (CD86, CCL2) and M2 marker genes (IL-10, CD206) in M1 macrophages after co-culture with Y-ASCs or O-ASCs. Data are presented as the mean ± SD (*n* = 3). Significance: *** *p* < 0.001, ** *p* < 0.01, * *p* < 0.05, ns: not significant.

To evaluate the age-dependent regulation of macrophage polarization by ASCs, we established and validated M0, M1, and M2 macrophage polarization models using RAW264.7 cells; flow cytometry and qPCR analysis of polarization markers confirmed the macrophage polarization models (Figure S2). M0 and M1 macrophages were co-cultured with Y-ASCs or O-ASCs, and surface marker expression was analyzed by flow cytometry. For M0 macrophages, Y-ASC co-culture significantly increased the proportion of CD206^+^ M2 macrophages (from 4.02% to 55.5%; *p* < 0.001) and had no effect on the proportion of CD11c^+^ M1 macrophages (Figure 2a). In contrast, O-ASC co-culture also significantly increased the proportion of CD206^+^ M2 macrophages (from 0.27% to 32.4%; *p* < 0.001) and had no effect on the proportion of CD11c^+^ M1 macrophages (Figure 2c). For M1 macrophages, Y-ASC co-culture effectively reversed their pro-inflammatory phenotype: CD206^+^ expression increased from 0.65% to 62.2%, CD11c^+^ expression decreased from 85.3 to 16.6 (*p* < 0.001; Figure 2b), while O-ASCs exerted minimal effects on M1 polarization, with significantly lower levels than those of Y-ASCs in CD11c or CD206 expression levels (Figure 2d). qPCR analysis of M1 markers (*Cd86*, *Ccl2*) and M2 marker genes (*Il-10*, *Cd206*) validated these findings: Y-ASC co-culture significantly downregulated the mRNA expression of *cd86* (by 2.1-fold; *p* < 0.01) and *ccl2* (by 1.8-fold; *p* < 0.05) and upregulated the mRNA expression of *IL-10* and *cd206* in M1 macrophages, while O-ASCs had no significant impact (*p* > 0.05; Figure 2e–h). These results demonstrate age-dependent immunomodulation by ASCs: Y-ASCs potently promote M2 polarization and suppress M1 polarization, whereas the polarization efficiency of O-ASCs on macrophages is significantly lower than that of Y-ASCs.

### 3.3. IL-1RA Expression Is Higher in Young ASCs than in Aged ASCs

To further elucidate the molecular mediators underlying this age-dependent immunoregulatory activity of ASCs, we analyzed the transcriptomic data of Y-ASCs and O-ASCs, then focused on interleukin-1 receptor antagonist (IL-1RA), a key anti-inflammatory cytokine implicated in macrophage polarization regulation, which was highly expressed in Y-ASCs (Figure S3). Moreover, serum cytokine analysis showed that Y-ASC transplantation reduced IL-1β levels by 42.3 ± 5.1% (*p* < 0.001 vs. aged mice), while O-ASCs had no effect (*p* > 0.05; Figure S4). To contextualize this age-dependent expression, we analyzed IL-1 family members in public datasets. GEO database analysis showed downregulated IL-1RA expression in ASCs from obese individuals, which is considered a model of accelerated metabolic aging, compared with normal-weight controls, accompanied by altered expression of IL-1 receptor subtypes (IL-1RI, IL-1RII, IL-1Racp) (Figure S3). Of note, obesity-related ASC changes do not fully recapitulate chronological aging, and this comparison serves only as supportive contextual evidence.

**Figure 3:**
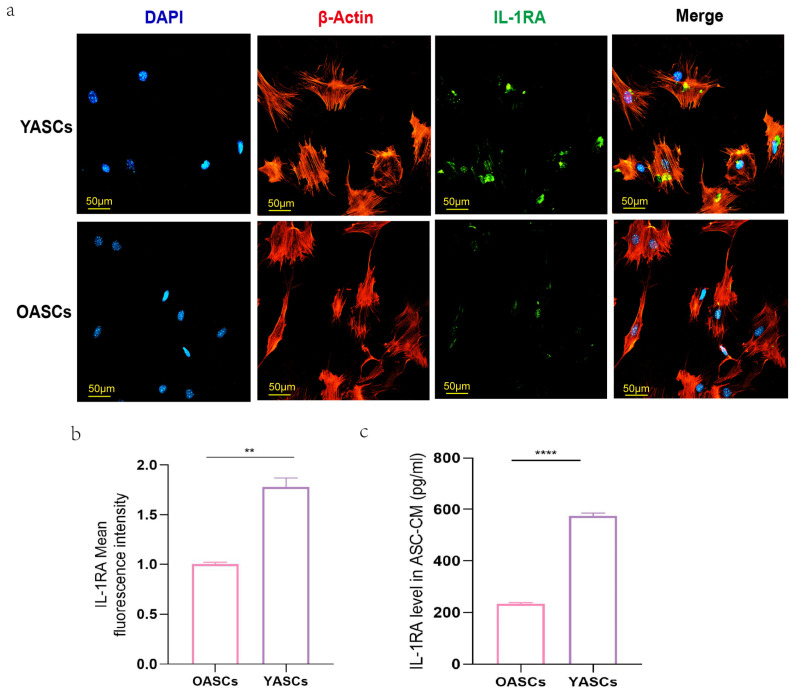
IL-1RA expression and secretion in Y-ASCs and O-ASCs. (**a**) Immunofluorescence images of IL-1RA (green), β-actin (red), and DAPI (blue) in Y-ASCs and O-ASCs (scale bar: 50 μm). (**b**) Quantitative analysis of the mean fluorescence intensity of IL-1RA. (**c**) ELISA measurement of sIL-1RA in ASC culture supernatants. Data are presented as the mean ± SD (*n* = 3). Significance: ** *p* < 0.01, **** *p* < 0.0001.

We next evaluated IL-1RA expression in ASCs to verify its age-dependent regulation by multiple approaches. Immunofluorescence assays showed that IL-1RA was predominantly cytoplasmic in both young (Y-ASCs) and aged (O-ASCs) ASCs, but Y-ASCs exhibited markedly stronger fluorescence signals (Figure 3a). Quantitative analysis confirmed that the mean fluorescence intensity of IL-1RA in Y-ASCs was higher than that in O-ASCs (*p* < 0.001; Figure 3b). ELISA further demonstrated that the secretion of soluble IL-1RA (sIL-1RA) in Y-ASC culture supernatants was significantly higher than that in O-ASC supernatants (*p* < 0.01; Figure 3c). These data collectively demonstrate that IL-1RA expression in ASCs is tightly regulated by donor age, with young donors exhibiting markedly higher levels.

### 3.4. IL-1RA Is a Critical Mediator of Y-ASC-Induced M2 Macrophage Polarization

**Figure 4:**
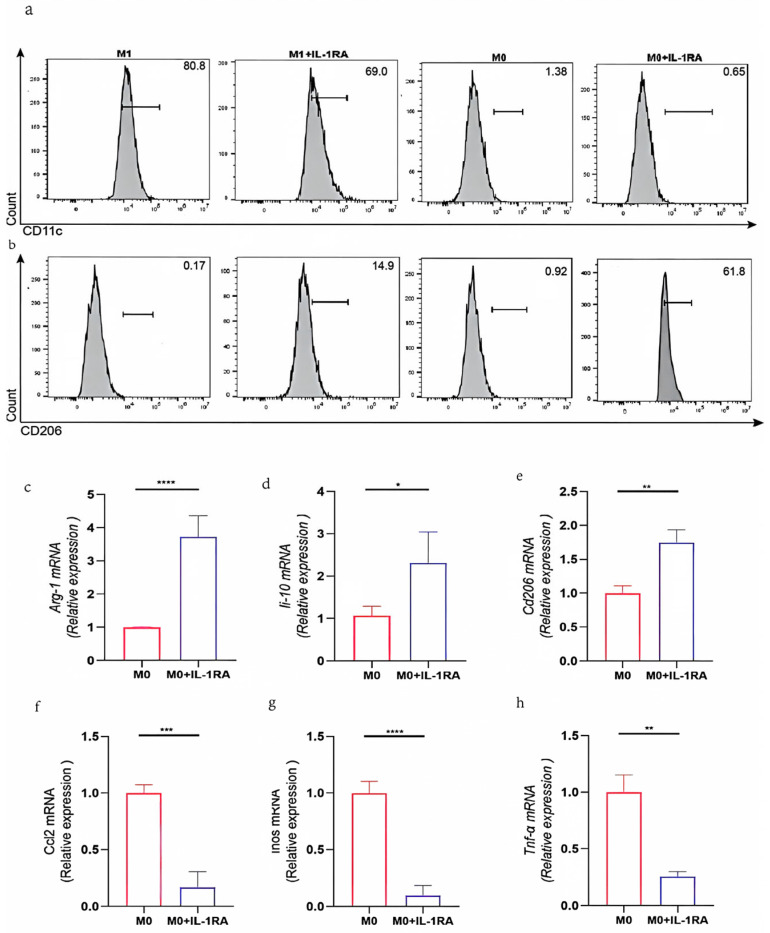
IL-1RA-mediated regulation of macrophage polarization. (**a**,**b**) Flow cytometric analysis of CD206 (M2 marker) expression in M0 and M1 macrophages treated with rIL-1RA. (**c**–**h**) qPCR analysis of M2 (Arg-1, CD206, IL-10) and M1 (Ccl2, iNOS, TNF-α) marker gene expression in macrophages treated with rIL-1RA. Data are presented as the mean ± SD (*n* = 3). Significance: **** *p* < 0.0001, *** *p* < 0.001, ** *p* < 0.01, * *p* < 0.05.

To confirm the role of IL-1RA in Y-ASC-mediated macrophage polarization, we performed gain-of-function and loss-of-function experiments. After treatment of M0 and M1 macrophages with recombinant IL-1RA (rIL-1RA), flow cytometry showed increases in CD206^+^ M2 macrophages (up from 0.92 to 61.8%; *p* < 0.001) and decreases in CD11c^+^ M1 macrophages (down from 80.8% to 69%; *p* < 0.05; Figure 4a,b). qPCR confirmed that rIL-1RA upregulated the mRNA expression of M2 markers (*Arg1*, *Cd206*, *Il10*) (*p* < 0.001) and downregulated the mRNA expression of M1 markers (*Ccl2*, *Nos2*, *Tnf*) (*p* < 0.01) in M0 macrophages (Figure 4c–h).

**Figure 5:**
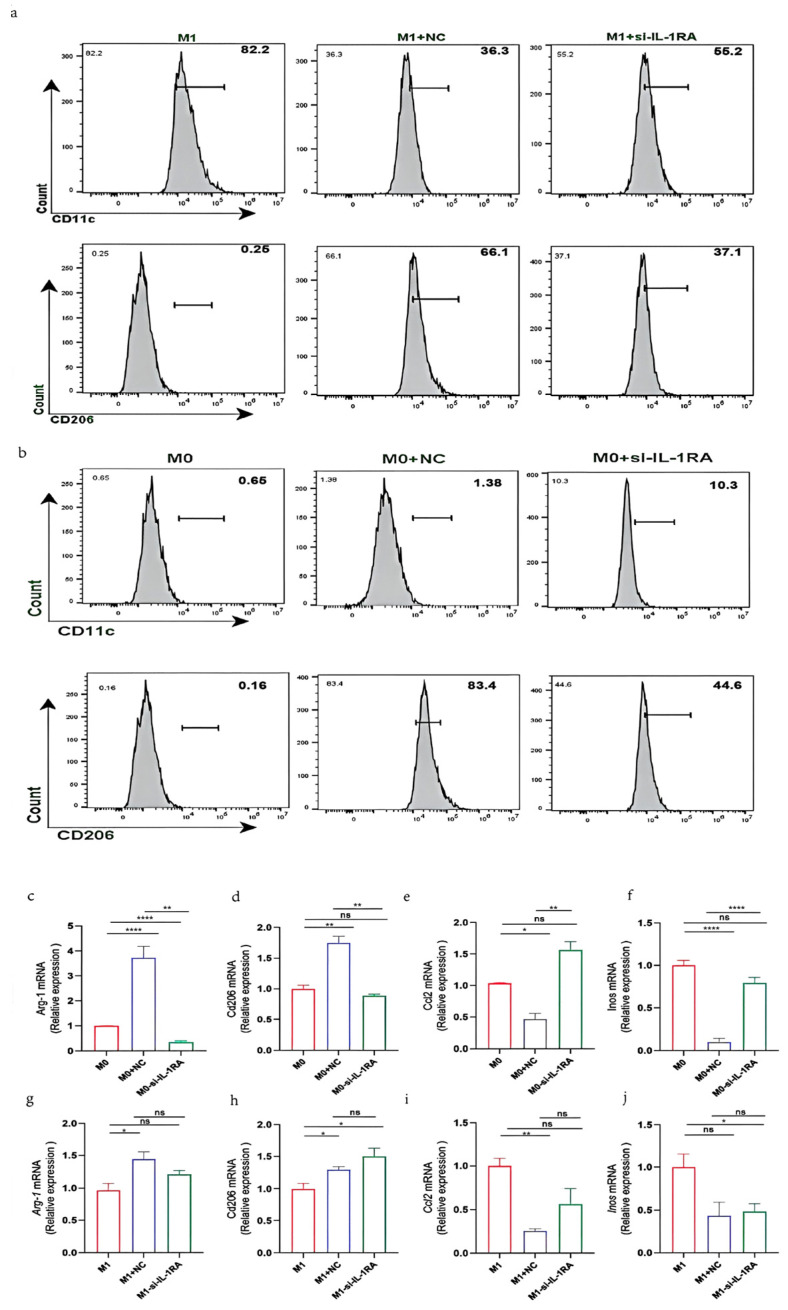
Effects of IL-1RA silencing in Y-ASCs on macrophage polarization. (**a**,**b**) Flow cytometric analysis of CD11c and CD206 expression in M0 and M1 macrophages co-cultured with si-IL-1RA-Y-ASCs. (**c**–**f**) qPCR analysis of M1 and M2 marker genes in M0 macrophages co-cultured with si-IL-1RA-Y-ASCs. (**g**–**j**) qPCR analysis of M1 and M2 marker genes in M1 macrophages co-cultured with si-IL-1RA-Y-ASCs. Data are presented as the mean ± SD (*n* = 3). Significance: **** *p* < 0.0001, ** *p* < 0.01, * *p* < 0.05, ns: not significant.

To validate the necessity of IL-1RA, we silenced IL-1RA expression in Y-ASCs via siRNA. ELISA and qPCR confirmed 82.3 ± 5.1% and 78.6 ± 4.8% reductions in IL-1RA mRNA and protein levels, respectively (*p* < 0.001; Figure 5). IL-1RA-silenced Y-ASCs (si-IL-1RA-Y-ASCs) did not affect M1 macrophage viability (*p* > 0.05) but abrogated anti-inflammatory activity: the Y-ASC-induced reductions in IL-1β and TNF-α secretion were partly reversed (vs. non-silenced Y-ASCs; Figure 5). Flow cytometry showed that si-IL-1RA-Y-ASCs failed to promote M2 polarization: the proportion of CD206^+^ M2 macrophages decreased from 66.1% (non-silenced Y-ASCs) to 37.1% (si-IL-1RA-Y-ASCs; *p* < 0.001) in M1, while the proportion of CD11c^+^ M1 macrophages was restored to 55.2 ± 4.1% (*p* < 0.001; Figure 5a). For M0 macrophages, si-IL-1RA-Y-ASCs co-culture failed to promote M2 polarization; CD206^+^ expression decreased from 83.4% to 44.6%, and CD11c^+^ expression increased from 1.38% to 10.3% (*p* < 0.001; Figure 5b). qPCR confirmed that IL-1RA silencing abolished Y-ASC-mediated regulation: M2 markers (Arg-1, CD206) were downregulated, and M1 markers (Ccl2, iNOS) were upregulated compared with non-silenced Y-ASC co-culture (*p* < 0.01; Figure 5c–j). These data establish IL-1RA as an essential mediator of Y-ASC-induced M2 polarization and anti-inflammatory activity in vitro.

## 4. Discussion

A core pathological hallmark of age-related metabolic disorders, such as obesity, is dysregulation of the adipose tissue immune microenvironment, which is characterized by excessive infiltration of adipose tissue macrophages and a pro-inflammatory milieu dominated by M1 macrophage polarization [2]. Regulating macrophage polarization balance is considered a promising strategy to delay aging and treat related diseases. Adipose-derived stem cells (ASCs) are promising candidates for immunomodulatory therapies, as their paracrine activity skews macrophage polarization toward the anti-inflammatory M2 phenotype [6]. Our previous research and other studies have confirmed that transplantation of Y-ASC significantly improves body weight and metabolic status in aged mice, whereas O-ASC does not [8,9], suggesting that donor age influences immune regulation and therapeutic outcomes. Although numerous studies have demonstrated the influence of ASCs on macrophage polarization [20], few have overlooked the correlation between the differences in transplantation efficacy across various ASC donor ages and their capacity to polarize macrophages. In this study, we demonstrated that Y-ASC transplantation reduced total macrophage abundance in aged mouse adipose tissue, decreased pro-inflammatory M1 proportions, and increased anti-inflammatory M2 populations. Similar results were also reported in a study on mesenchymal stem cells (MSCs), suggesting that young mouse MSCs induced M1 apoptosis, suppressed inflammatory cytokine production by M1, and induced macrophage polarization toward M2 [21]. In vitro Y-ASC and O-ASC co-culture with RAW264.7 cells observed results consistent with those in vivo. However, the mechanisms of Y-ASC and O-ASC with macrophage polarization are not completely understood.

In in vivo transplantation experiments, we also observed that Y-ASC transplantation reduced serum IL-1β levels in aged mice, while O-ASCs had no significant effect. IL-1β is a key driver of inflammaging and age-related metabolic dysfunction [22,23]. Owing to the central role of IL-1 in the pathogenesis of inflammation-driven diseases, IL-1 blocking agents are approved for clinical use in several inflammatory conditions [23]. Studies have pointed out that MSC exosomes induced by IL-1β enhance anti-inflammatory activity [24,25]. Transcriptomic data revealed significant differences in interleukin-1 receptor antagonist (IL-1RA) expression between Y-ASC and O-ASC. IL-1RA, a natural antagonist of the IL-1 signaling pathway, functions as a negative regulator by blocking IL-1β-induced pro-inflammatory signals [23]. Studies have demonstrated that mesenchymal stem cell-derived IL-1RA promotes macrophage polarization to M2 [25]. Our results confirmed that Y-ASCs exhibit markedly higher IL-1RA expression and secretion than O-ASCs, a difference directly correlating with their superior ability to regulate macrophage polarization. Recombinant IL-1RA (rIL-1RA) recapitulated the M2-polarizing and anti-inflammatory effects of Y-ASCs, while siRNA-mediated IL-1RA silencing largely abrogated these effects, partially restoring M1 proportions and inflammatory cytokine secretion. This firmly establishes IL-1RA as an essential mediator of Y-ASC-induced immunomodulation in vitro. In O-ASCs, reduced IL-1RA expression may disrupt this homeostatic balance, perpetuating chronic inflammation and impairing therapeutic efficacy as observed in our in vivo transplantation experiments. This framework not only partially explains Y-ASCs’ superior performance but also identifies actionable strategies to optimize ASC-based therapies for age-related inflammatory disorders.

Beyond confirming IL-1RA’s central role, our results uncovered a notable novel finding about IL-1RA’s selective involvement in macrophage polarization. Specifically, IL-1RA silencing primarily impaired M0-to-M2 polarization rather than M1-to-M2 conversion, suggesting it acts at the early stage of macrophage differentiation. Different phenotypes of macrophages (M0, M1 and M2) have been demonstrated to play distinct roles in regulating mesenchymal stem cells; M1 supported the proliferation and adipogenic differentiation of bone marrow mesenchymal stem cells, whereas M0 or M2 showed an enhanced effect on cell osteogenic differentiation [26]. This suggests that different macrophage subtypes can influence the adipogenic and osteogenic differentiation of ASC, potentially representing a key mechanism underlying weight loss following ASC transplantation. This observation deepens our mechanistic understanding and implies M1 reprogramming may depend on additional factors (e.g., cell–cell contact, IL-10, TGF-β) [27], highlighting a complex, multi-faceted regulatory network in ASC–macrophage crosstalk worthy of further exploration.

However, this study has several limitations that should be acknowledged. First, our in vitro macrophage findings relied on an immortalized cell line and a focused phenotypic panel, which warrants broader validation in primary immune cells. Second, the study included only male mice, which limits the generalizability of the findings to both sexes, and sex as a biological variable should be investigated in future studies. In spite of these considerations, the precise in vivo pathways connecting Y-ASC efficacy to IL-1RA remain to be fully confirmed through direct loss-of-function or rescue models. Furthermore, without longitudinal cell-tracking and biodistribution data following intraperitoneal injection, the exact mode of inter-organ communication remains to be definitively charted. Finally, systemic inflammatory profiling focused primarily on serum IL-1β, leaving room for a more expansive cytokine and metabolic evaluation.

## 5. Conclusions

In summary, our in vitro study demonstrates that IL-1RA serves as an important age-sensitive mediator of ASC-induced immunomodulation, promoting M2 macrophage polarization and attenuating chronic inflammation via reducing IL-1β levels. Notably, diminished expression and functional activity of IL-1RA in O-ASCs may weaken this regulatory cascade. The findings provide mechanistic insights into the age-dependent functional decline of ASCs and may facilitate the translation of ASC-based therapies for age-related metabolic and inflammatory disorders.

## Data Availability

The data presented in this study are openly available on the China National Center for Bioinformation/Beijing Institute of Genomics’ website at https://ngdc.cncb.ac.cn/gsa (reference number CRA008155).

## Supplementary Materials

The following supporting information can be downloaded at: https://www.mdpi.com/article/10.3390/cells15151355/s1, Figure S1: Characterization of M0, M1, and M2 macrophage polarization using RAW264.7 cells; Figure S2: IL-1 family expression profiles in aged and obese models; Figure S3: Age-related changes in serum IL-1β levels; Figure S4: Effects of IL-1RA silencing in Y-ASCs on macrophage function; Table S1: qPCR Primers.
